# A new perspective on the interplay between self-control and cognitive performance: Modeling progressive depletion patterns

**DOI:** 10.1371/journal.pone.0180149

**Published:** 2017-06-29

**Authors:** Christoph Lindner, Gabriel Nagy, Wolfgang Andreas Ramos Arhuis, Jan Retelsdorf

**Affiliations:** Leibniz Institute for Science and Mathematics Education at Kiel University, Kiel, Germany; Radboud Universiteit, NETHERLANDS

## Abstract

Exerting self-control in a first task weakens self-control performance in a subsequent unrelated task (ego depletion). In self-control research new strategies are required to investigate the ego-depletion effect, which has recently been shown to be more fragile than previously assumed. Moreover, the relation between ego depletion and trait self-control is still unclear, as various studies have reported heterogeneous findings concerning the interplay of both variables. We addressed these lacunas by drawing on a sample of *N* = 120 students, who participated in two test sessions. In the first test session, we assessed trait self-control and several control variables. The second test session followed an experimental design and tested the effects of ego depletion on invested effort and cognitive performance trajectories in an ecologically valid computer-based assessment setting (i.e., a 30-minute mathematical problem-solving and reasoning test). Trait self-control was then used as a moderator of the ego-depletion effect. Combining an established ego-depletion paradigm (i.e., the sequential-task paradigm) with multilevel modeling of time-on-task and performance changes, our results indicate (1) that trait self-control predicted the motivation to solve cognitive tasks, (2) that ego depletion led to a progressive performance decrease, and (3) that the negative effect of ego depletion on performance was stronger for students with high trait self-control. Additional analyses revealed that our results could not be alternatively explained by fatigue effects. All effects were robust even after controlling for the students’ cognitive abilities, which are known to be closely related to mathematical performance. Our results provide evidence that the self-control invested in order to keep performance at a consistently high level wanes over time. By modeling progressive ego-depletion effects while considering trait self-control, we provide an alternative approach that may help future researchers to investigate the underlying mechanisms of self-control.

## Introduction

*Self-control* refers to the cognitive processes that enable people to override, inhibit or modify their impulses, thoughts, emotions and behavior and to bring them into line with standards and personally endorsed overarching goals [1,2]. Furthermore, self-control is necessary in order to be able to consistently direct attention toward relevant information when working on reasoning or mathematics tasks [3, 4], while shielding information processing from interferences such as task-irrelevant thoughts [5]. Although various experimental studies (for an overview see [3]) found that the exertion of self-control impairs individuals’ performance on subsequent tasks that also require self-control or cognitive processing, the underlying mechanisms of this so called *ego-depletion effect* are still unclear.

In connection to recent discussions that the development of ego-depletion effects might be more complex and fragile than expected [6], several authors (e.g., [2, 6, 7, 8]) have suggested that the ego-depletion phenomenon needs to be explored in greater detail, while considering variables (e.g., trait self-control) that seem to moderate the ego-depletion effect [9].

With the aim of achieving a better understanding of the ego-depletion effect, the present study follows these long-standing calls for a deeper investigation of ego-depletion-dependent processes, such as progressive performance changes that occur when working on tasks that require self-control in order to keep attention focused during information processing (e.g., [8,10]), in an ecologically valid assessment setting, while considering trait self-control as a major predictor of achievement-related outcomes (e.g., [11, 12]. It should be noted that the heterogeneous findings on the interplay between ego depletion and trait self-control (for an overview see [13]) need to be clarified in greater detail [9] in order to gain deeper insights into the underlying mechanisms of self-control.

For our investigation, we used a well-established ego-depletion manipulation paradigm while modeling time-on-task and performance changes over the course of a computer-based mathematical problem-solving and reasoning (MPSR) test, while accounting for differences in trait self-control. In doing so, we provide a strategy for investigating both the average and the progressive effects of ego-depletion and trait self-control in a cognitive task that requires self-control in order to keep attention consistently focused on the items’ content while shielding information processing from interferences. Our approach goes beyond the traditional approach taken in ego-depletion research, which restricts its focus to individuals’ average task performance when they are in a state of ego-depletion. Furthermore, we also examined whether ego depletion, trait self-control, and their interaction affect students’ test-taking motivation and fatigue. Our research thus provides some new insights into the role that self-control plays in achievement-related behavior and addresses current hot topic questions in self-control research [10].

### Ego depletion and its relation to trait self-control

Two prominent theories have been proposed to explain the ego-depletion phenomenon. First, the *strength model of self-control* [1, 14] postulates that any process requiring active self-control leads to a short-term depletion of a finite domain-independent mental resource (i.e., ego depletion). One consequence of ego depletion is that fewer resources are available for subsequent activities requiring self-control or working-memory operations (e.g., [15]). To illustrate their strength model, Baumeister, Vohs and Tice [1] postulated that the depletion of self-control resources resembles muscle fatigue—i.e., muscle performance decreases after sustained physical exercise and is restored after rest. Over the last 20 years, various studies have confirmed the ego-depletion effect in different experimental settings and have explained the performance decrements by referring to the postulated short-term lack of self-control resources [3]. Especially younger adults [16] but also school children (e.g., [17, 18, 19, 20]) seem to be susceptible to ego depletion and its aftereffects. However, despite these findings a general discussion about the robustness and even the existence of the ego-depletion effect has recently evolved [6].

Second, due to an emerging number of empirical findings that seem to be incompatible with the strength model (e.g., [21, 22, 23], Inzlicht and Schmeichel [8] provided an alternative explanation for ego-depletion effects. In their *process model of ego-depletion*, they suggest that self-control exertion becomes more and more aversive over time, leading to a disengagement from effortful tasks that require self-control (cf. [24]), while attentional shifts toward reward-related stimuli increase. As a consequence of attentional disruptions and reduced effort, individuals’ performance in problem-solving tasks or cognitive performance tests decreases (e.g., [4]). Following the process model, ego depletion is assumed to be reflected in attentional shifts and reduced effort, which is the lack of motivational intensity invested to carry out instrumental behavior [25]. To further investigate Inzlicht and Schmeichel’s assumption about the underlying mechanisms of ego depletion [8], alternative methods are needed to test ego-depletion-dependent changes in effort and performance that occur when working on tasks that require self-control in order to shield cognitive processing from distractions (cf. [10]). Up until now, ego-depletion induced motivational and performance changes over the course of a cognitive test have not been investigated in self-control research.

Another topic in self-control research that has only been poorly understood up until now concerns the effects of the interplay between ego depletion and trait self-control on performance and achievement-related behavior (cf. [9, 13]). Trait self-control is assumed to be positively related to effortless strategies that individuals use for dealing with self-control dilemmas [13]. However, contrary to the long-standing assumption that high trait self-control protects individuals from ego-depletion effects, (e.g., [26]), Stillman, Tice, Fincham and Lambert [27] did not find a moderating effect of trait self-control on the relation between ego depletion and mathematics performance. Imhoff, Schmidt and Gerstenberg [9] even found a counterintuitive relation between trait self-control and ego depletion: Individuals with high trait self-control were less able to resist tempting food, showed more risk-taking behavior and less achievement motivation when they were in a state of ego depletion, compared to individuals with low trait self-control in a state of ego depletion. Furthermore, they found that individuals with high trait self-control engaged in fewer everyday activities that require self-control and even seemed to use effective strategies to avoid situations that require self-control and might lead to ego-depletion effects in the first place. Thus, the authors speculate that people with high trait self-control might have less practice in dealing with self-control conflicts in ego-depletion experiments; therefore, they show stronger ego-depletion effects than low trait self-control people. To clarify this counterintuitive interplay between trait self-control and ego depletion in more detail, Imhoff et al. [9] suggested examining the effect of the interaction between trait self-control and ego depletion on tasks that require the initiation of self-control in order to persevere and to keep performance on a consistently high level over the course of time.

We aimed to add to the research on ego-depletion by addressing the two aforementioned neglected issues. Therefore, we used an experimental, ecologically valid setting to test the effects on dependent variables that require self-control for cognitive processing over a longer period of time and that should be susceptible to the ego-depletion effect as well as to trait self-control. Thus, we investigated how ego depletion and trait self-control affected two aspects of a school achievement test (i.e., MPSR test): test-taking effort (i.e., time-on-task) and performance (i.e., probability of correct item responses). Achieving good performance on such tests is assumed to require high amounts of self-control for effective information processing and, therefore, seems to be susceptible to ego-depletion effects (e.g., [28, 29, 30]). In the following, we briefly review previous research on the relevance of trait self-control and ego depletion for achievement testing before we describe our own research in more detail.

### Self-control in achievement testing

Working on achievement tests requires the exertion of self-control in order to initiate cognitive processing and focus attention on the test content while inhibiting distractions. The executive functions (i.e., working-memory updating, inhibiting and shifting) [31] are key ingredients for successful self-control [5]. They seem to play a crucial role in inhibiting attentional drifts toward distractors, but also in updating test-relevant information in working memory, and in shifting between tasks or mental sets [5, 31]. Although the underlying mechanisms of the ego-depletion effect are still unclear, there has been compelling support for the assumption that ego depletion negatively affects students’ test-taking effort and cognitive performance: Individuals in a state of ego depletion were less successful in solving anagram tasks (e.g., [32, 33]) and performed worse than non-depleted individuals in working-memory [15], logical reasoning [4], and mental arithmetic tasks (cf. [3]). It should be noted that working memory and reasoning abilities are also key ingredients in the context of students’ problem solving in mathematics (e.g., [34, 35, 36]). A few studies (e.g., [17, 28]) indicate that students in a state of ego depletion have fewer mental capacities available to keep their attention consistently focused to operate on stored information when working on mathematical tasks (cf. [37]), while simultaneously inhibiting any distracting thoughts or emotions. This is in line with the findings of Englert and Bertrams [18], who showed that eighth-graders’ knowledge retrieval was undermined only when the students previously had to invest self-control to control their writing habits and therefore became ego depleted. The authors point out that ego depletion might negatively affect academic performance in school children.

Moreover, psychometric research suggests that students’ performance decreases that occur during achievement testing (e.g., [38, 39, 40]) might depend on fluctuations in executive attention [41], which, in turn, is assumed to be related to ego-depletion effects and to the effectiveness of working memory operations (e.g., [5]). Thus, in the present study, we additionally addressed the open question of whether these performance decreases depend on ego-depletion effects. We expected to find worse performance and larger performance decreases over the course of testing for depleted compared to non-depleted students. Moreover, we expected to find larger decreases in the time invested by depleted versus non-depleted students, as previous studies found that ego depletion undermines persistence (i.e., less time-on-task) and stamina in tasks that require self-control (e.g., [32, 33, 42]).

Alongside the possible negative ego-depletion effects on performance and achievement behavior, trait self-control has been positively related to students’ test performance [43, 44]. This relation may be explained by the assumption that trait self-control determines the effort invested to solve test items [45]. Effort investment seems to be positively related to time-on-task in tests that require reasoning [46], indicating that high-trait self-control individuals, who are assumed to use more effortless strategies [13] to control their attention and thoughts, might show a stronger engagement in time-consuming, controlled information-processing when focusing on problem-solving tasks. Since trait self-control is also assumed to be positively related to persistence in self-controlled behavior over longer periods of time [47], it might be positively related to time-on-task and performance throughout testing.

Considering trait self-control and ego depletion together in the present research makes it possible to investigate their interplay. As described above, it is not yet clear how these variables interact (cf. [13]). Therefore, we examined the effects of the interplay between trait self-control and ego depletion on time-on-task and test performance as an exploratory research question.

### The present study

Our study had two main goals: First, we aimed to investigate ego-depletion-dependent processes, such as motivational and performance changes, over the course of time (cf. [8, 10]) in an ecologically valid assessment setting, while considering trait self-control and its interaction with ego depletion. Since a variety of studies found that ego depletion briefly undermines participants’ persistence (e.g., [32, 33, 42]) as well as their information processing (cf. [3]) and reasoning abilities [4, 15] in mathematics (e.g., [3, 28, 37]), our dependent variables were time-on-task as an indicator of the invested test-taking effort (i.e., the motivation to achieve good performance) and test performance over the course of a 30-minute MPSR test. We expected depleted students to spend less average time and show worse mean performance on the MPSR test than non-depleted students. Moreover, we expected the ego-depletion effect to be reflected in larger decrements in time-on-task and performance over the course of testing.

Since high-trait self-control individuals seem to use effortless strategies for dealing with self-control demands [13], we assume that they are highly motivated to achieve good performance on cognitive tasks, which they might handle more easily than low-trait self-control individuals. Therefore, we expected trait self-control to be positively related to time-on-task, performance, and the stability of both remaining on a consistently high level over the course of testing. Furthermore, we tested the interaction effects of trait self-control and ego depletion on time-on-task and performance without any specific hypotheses since previous research has provided heterogeneous findings on this relation (for an overview cf. [13]).

Second, we aimed to apply an alternative approach to investigating ego-depletion-related processes over the course of time. Therefore, we combined the traditional sequential-task paradigm (i.e., students work on two consecutive tasks that require self-control) with multilevel regression modeling. This made it possible to test how ego depletion, trait self-control, and their interaction affect students’ average time-on-task, test performance, and the development of both throughout testing. To the best of our knowledge, our study is the first to investigate ego-depletion effects on achievement tests while considering changes in the invested test-taking effort and performance over the course of testing.

Finally, we also tested whether the ego-depletion manipulation induced fatigue effects or changes in further relevant covariates such as self-reported motivation and affect.

## Materials and methods

### Ethics statement

The protocol of the present study was discussed at and ethically approved by the research colloquium of the Leibniz Institute for Science and Mathematics Education. Prior to both test sessions, we obtained written informed consent from all the participants and their parents. This study was carried out in accordance with the Declaration of Helsinki and the ethical guidelines for experimental research with human participants as proposed by the German Psychological Society (DGPs) and the American Psychological Association (APA).

### Participants

We determined the total sample size needed to fulfill the requirements of our multilevel basic model (M0), which is equivalent to the ANOVA test that is traditionally used for investigating mean performance differences between the depletion and control group. Using G*Power [48] and assuming a medium effect (*f* = .25) of ego depletion on mathematical performance (cf. [3]), an *N* of 128 is needed for 80% power. This sample size was also sufficient for estimating the multilevel models used in this study [49].

Since previous studies [17, 18, 19, 20] have found that school children’s academic performance is undermined when they are in a state of ego depletion, we recruited 129 tenth graders from various classes of six comprehensive schools in Germany to participate in two standardized test sessions carried out at the Leibniz Institute for Science and Mathematics Education at Kiel University. All participants were compensated with €20 for two test sessions. Seven students failed to follow the instructions for the experimental ego-depletion manipulation and two students reported health issues and that they were not able to concentrate on the MPSR test. Thus, nine students were excluded, leading to a final sample of *N*= 120 students (57 female; *M*_*age*_= 15.7, *SD*= 0.71); we randomly assigned 59 students (26 female) to the *ego-depletion group* (EDG) and 61 students (31 female) to the *no depletion group* (NDG) of the between-group design. None of the participants guessed the purpose of our study.

### Procedure, measures and experimental material

In order to ensure that the EDG and NDG did not differ in various achievement-related characteristics, we assessed control variables (i.e., cognitive abilities, grades, and self-concepts) and trait self-control in a first test session a few weeks before the main experiment (test session two) took place. As a cover story, all participants were told that we were investigating differences in their achievement-related behavior when they were working on paper-and-pencil (test session one) versus computer-based tests (test session two). Furthermore, we asked them to perform as well as possible in both test sessions. In the following, we describe the procedure of both test sessions in detail.

#### Test session one

All students participated in standardized group test sessions and were seated two seats apart from each other in the lecture hall. After a general introduction, the participants completed the subtests *Word Analogies*, *Numerical Series* and *Figure Analogies* of the German adaptation of the Cognitive Abilities Test [50]. We used the overall score to measure general cognitive abilities. Next, *gender*, *age* and *latest report card grades* (in mathematics, science, German, and English) were assessed, followed by *general academic self-concept* (four items; e.g., “Compared to others, I am less gifted.”) and *mathematical self-concept* (four items; e.g., “I am good in mathematics.”), using a German adaptation of the Self-Description Questionnaire III [51]. The students had to react to the statements on a 4-point Likert scale, anchored at 1’*not at all*’ and 4 *‘absolutely’*. Finally, we measured *trait self-control* using a German version of the Brief Self-Control Scale [52]. All 13 items (e.g., “I say inappropriate things.”) were rated on a 5-point Likert scale, anchored at 1 ‘*not at all like me’* and 5 ‘*very much like me’*.

#### Test session two (Main experiment)

In each of the computer-based test sessions, up to six participants were seated at separate computer terminals which were equipped with identical hardware and software components. Thus, all participants worked on their own and were not able to copy from each other. We used the PHP-framework *flexSurvey* [53] to implement the tests and questionnaires and we provided the content on a local webserver (*XAMPP*; [54]). After a general introduction, the participants rated their *state of fatigue* from 1 ‘*not at all’* to 5 *‘very’* (six items; e.g., “How do you feel at the moment?”—tired) on the awake-tired subscale (α = .90) of the Multidimensional Mood State Questionnaire [55]. Since weak ego-depletion effects on performance might be due to unfulfilled requirements for overriding habits during the ego-depletion manipulation task [56, 57, 58], we applied a handwriting task as a well-established and valid ego-depletion manipulation (cf. e.g., [17, 28, 58, 59, 60]) that has also been used to investigate ego-depletion effects in school children (e.g., [18]). All students were instructed to transcribe a text about the history of the city of Mannheim, Germany. Whereas no further instructions were given to the NDG, students in the EDG were additionally instructed to control and inhibit their writing habits by omitting any instances of the most frequently used letters in German: *e* and *n*. For example, instead of “Mannheim”, the students had to write “Mahim”. Writing texts at school is part of students’ daily routine, thus, EDG participants had to exert self-control on their handwriting habits by inhibiting impulses to write the full words and, instead, omitting the forbidden letters. After 8 minutes, the test administrator stopped the transcription task and all students answered items from the manipulation test as proposed by Bertrams et al. [18, 28]. More precisely, the degree of self-control exerted on the handwriting task (four items; e.g., “To what extent did you suppress your normal writing habits in this transcription task?”) was assessed on a 7-point Likert scale, ranching from 1 ‘*not at all’* to 7 *‘very’*. To ensure that the manipulation did not induce changes in the students’ affect, we applied the German version of the Positive and Negative Affect Schedule [61]. The participants rated their *positive* (ten adjectives; e.g., “attentive”) and *negative affect* (ten adjectives; e.g., “angry”) on a 5-point Likert scale, ranging from 1 *‘not at all’* to 5 *‘extremely’*. Furthermore, we examined ego-depletion-induced motivational changes by assessing the *intrinsic motivation* to perform well in the following computer-based tasks using three items (e.g., “How motivated are you at the moment to work on the following tasks?”). Two *mathematics motivation* items were used to measure the degree of motivation to perform well in the upcoming MPSR test (e.g., “How much effort will you invest when working on the following mathematics task?”). All motivation items were rated on a 7-point Likert scale, anchored at 1 *‘not at all’* and 7 *‘very’*.

Afterwards, all students worked on a computer-based multiple-choice test in mathematics. All items (difficulties ranged from medium to complex) were taken from the German National Educational Standards in mathematics for tenth graders [62] and measure mathematical reasoning, problem solving, and modeling. The items were presented in a fixed order and the students could achieve a maximum of 45 points (i.e., one point for each item). Missing responses were scored as incorrect. We additionally analyzed our data by defining missing responses as missing values. The results were basically the same. The students were not given any information about the test length and time, had to select an answer to each item, and could neither skip backwards to previous items nor forwards to subsequent items. The students were informed that the test was designed for tenth graders and that they should try to solve as many items as possible. Students who finished the test early were given a dummy writing task. After 30 minutes, all computer screens switched synchronically to the final questions. The students rated their state of fatigue on the awake-tired subscale again (see above) and rated the effort they had invested for solving the items in the mathematics test compared to a maximum effort they would invest in a situation that is of high personal importance [63].

### Data analyses

To investigate the effects of trait self-control and ego depletion on time-on-task and performance over the course of the MPSR test, we applied multilevel regression models [64] using M*plus* 7.0 [65]. These models served two purposes. First, the models assessed within-student changes in processing time and the probabilities of correct responses over the sequence of items presented in the MPSR test. More specifically, the models assessed whether processing times and the probabilities of correct responses showed a systematic trend from the beginning to the end of the test. This feature is represented in the Level 1 equations, which can also be conceived as the within-student part of the model.

The second purpose of the models employed was to relate individual differences in average response times and probabilities of correct item responses, as well as individual differences in changes in processing times and correct responses assessed over the course of the MPSR test to student characteristics, including the ego-depletion treatment condition and a measure of trait self-control. This feature is included in the Level 2 part of the model that refers to the between-student level. Hence, in combination with the Level 1 part, the Level 2 part made it possible to investigate whether, for example, students in the two ego-depletion treatment conditions (i.e., EDG vs. NDG) differed significantly from each other in their average response times and average probabilities of correct item responses (i.e., the main effects of the treatment conditions). Furthermore, the models made it possible to examine whether the treatment effect changed over the course of the MPSR test (i.e., the interaction effects of the treatment conditions with the item positions in the test); an interpretation that would be indicated by statistically significant differences in changes in response times and probabilities of correct responses between treatment groups.

For example, in the case of processing time, *y*_*ij*_ stands for the natural logarithm of the seconds spent by individual *i* (*i* = 1, 2, …, *N*) on item *j* (*j* = 1, 2, …, *J*). In order to study whether processing time showed a systematic trend over the course of the MPSR test, processing time was regressed on the position of the item *j* in the test that was coded by the variable *p*_*j*_. The values of *p*_*j*_ ranged from -0.5 in the first position to +0.5 in the last position of the test. We chose this particular coding of positions because it allows for a straightforward interpretation of regression coefficients (see below).

The Level 1 model for the outcome variable ‘processing time’ took the following form:

$$y_{ij}=\gamma_{0i}+\gamma_{1i}p_{j}+\sum_{j=1}^{J}\gamma_{j}d_{j}+\varepsilon_{ij},$$ (1)

where the parameters *γ*_0*i*_ and *γ*_1*i*_ are of key interest. Note that these parameters are indexed by the person subscript to indicate that they were allowed to take different values for each student in the sample. *γ*_0*i*_ is an intercept parameter, specific to individual *i*, which captured student *i*’s average response time (adjusted for the impact of the covariates *d*_*j*_; see below), so that higher values of *γ*_0*i*_ imply higher average response times. *γ*_1*i*_ is an individual specific slope parameter representing the effect of the item position on the time-on-task (adjusted for the covariates *d*_*j*_). The value of *γ*_1*i*_ reflects the individual *i*’s difference in time-on-task spent at the end versus the beginning of the test. Similar to the intercept term, this coefficient was allowed to vary across individuals, which means that the model acknowledged that the discrepancy in time-on-task between the end and the beginning of the MPSR test could differ between individuals.

As shown in Eq 1, the Level 1 model includes an additional set of covariates (the *d*_*j*_-terms) that are accompanied by a set of regression weights (*γ*_*j*_-coefficients). The *d*_*j*_-terms stand for a set of dummy variables that were used to identify each item *j*, so that a value of *d*_*j* = *k*_ = 1 indicated that the outcome variable was measured with respect to item *j* = *k*, and *d*_*j* = *k*_ = 0 indicated otherwise. Hence, the *γ*_*j*_ parameters in Eq 1 captured item effects that accounted for the possibility that some items could require more processing time than others. Finally, *ε*_*ij*_ is a residual (or error) term with zero expectation.

The main purpose of the Level 1 model presented in Eq 1 was to define the person-specific intercept (*γ*_0*i*_; *i*’s average time spent on the test items) and the slope parameters (*γ*_1*i*_; difference in time-on-task spent at the end versus the beginning of the test). These parameters were allowed to vary between students on the Level 2 part of the model (i.e., the between-student part), in which they were related to student-level covariates. Hence, the Level 2 model made it possible to examine whether individual differences in average response times (i.e., intercepts *γ*_0*i*_) and changes in response times (i.e., slopes *γ*_1*i*_) could be predicted by student-level characteristics. To this end, both parameters were related to *K* (*k* = 1, 2, …, *K*) person variables *x*_*k*_ by the following equations:

$$\gamma_{0i}=\alpha_{0}+\beta_{01}x_{i1}+\cdots +\beta_{0K}x_{iK}+\zeta_{0i}\;,$$ (2)

and

$$\gamma_{1i}=\beta_{11}x_{i1}+\cdots +\beta_{1K}x_{iK}+\zeta_{1i}.$$ (3)

In Eq 2, *α*_0_ stands for a structural intercept parameter, whereas the *β*_0*k*_ parameter and *β*_1*k*_ parameter in Eq 3 describe the effects of the individual covariates on the intercept (Eq 2) and the slope parameters (Eq 3), and *ζ*_0*i*_ and *ζ*_1*i*_ stand for residual terms with zero means. Note that Eq 3 does not include a structural intercept term (i.e., *α* parameter) because the data only allowed individual differences in within-student effects of item positions (*p*_*j*_ in Eq 1) to be uniquely identified (i.e., the structural intercept was absorbed in the *γ*_*j*_ parameters of Eq 1). However, the model did facilitate an estimation of the effects of all the covariates considered on the individual differences in the change in time-on-task. As such, the model made it possible to examine whether changes in time-on-task differed between the EDG and the NDG. For example, in the case of Eq 2, a statistically significant *β*-coefficient associated with the treatment indicator would indicate a main effect of the treatment because the outcome variable represents the average time-on-task. In contrast, in the case of Eq 3, a significant *β*-coefficient would indicate an interaction effect between the ego-depletion treatment condition and the items’ positions in the test, thereby providing evidence that the treatment effect shows a gradual change over the course of the test-taking situation. Furthermore, as shown in Eqs 2 and 3, the Level 2 model is flexible inasmuch as it allows many predictor variables to be considered, including interaction terms such as the interaction between the ego-depletion treatment condition and trait self-control, among others.

Up to this point, we have only presented the multilevel model that was applied to processing time. The setup of the model applied to the probabilities of correct item responses was similar to the model given in Eqs 1 to 3. However, as item responses were dichotomously scored, the dependent variable in Eq 1 was replaced by the logit of the probability of a correct item response: log[*P*(*y*_*ij*_ = 1)/*P*(*y*_*ij*_ = 0)], with *y*_*ij*_ = 1 indicating a correct and *y*_*ij*_ = 0 an incorrect response of individual *i* to item *j*.

The models outlined in Eqs 1 to 3 were specified by recurring on different between-student variables, including a dichotomously scored ego-depletion treatment indicator (NDG = 0; EDG = 1), a trait self-control score, and the interaction between ego-depletion treatment and trait self-control. All continuous variables on the between-student level were z-standardized prior to the analysis to facilitate the interpretation of results. Finally, for the probabilities of solving the items, the associated standard errors of estimates were computed by means of the delta method. This procedure enabled the calculation of confidence intervals for the item solving probability for the EDG in reference to the NDG.

## Results

### Randomization and manipulation

The EDG and NDG did not differ in control variables, thus our randomization succeeded, as did the ego-depletion manipulation (see Table 1).

**Table 1:** Randomization and manipulation checks using unpaired t-tests.

Group		*Ego-Depletion N* = 59	*No Depletion N* = 61			
	*α*	*M (SD)*	*M (SD)*	*d*	*t*(118)	*p*
Cognitive abilities	.84	37.63 (8.48)	36.13 (8.89)	0.17	0.94	.348
Report card grade in math	-	3.15 (1.06)	3.12 (1.04)	0.03	1.17	.853
Grade point average	-	2.86 (0.68)	2.77 (0.72)	0.13	0.72	.473
Mathematical self-concept	.80	11.17 (2.61)	11.33 (2.78)	0.06	- 0.32	.748
Academic self-concept	.80	12.49 (2.58)	12.41 (2.72)	0.03	0.17	.866
Trait self-control	.79	43.14(7.78)	40.89 (8.09)	0.28	1.55	.123
Manipulation check	.71	16.59 (4.97)	11.59 (4.13)	1.10	6.00	< .001

*Note*. *α* = Cronbach’s reliability coefficient. *d* = Cohen’s effect size. All *t*-tests were two-tailed.

### Self-control and Time-on-task

Due to the dummy-coded treatment variable, the corresponding results in the models T0 and T1 (see Table 2) can be interpreted as effects on the time-on-task (in logtime) for the EDG in reference to the NDG. The models’ intercept represents the average time students spent working on the test when they were assigned to the EDG in reference to the NDG, whereas the effects on the slope represent changes in time-on-task for the EDG in reference to the NDG. Contrary to our assumption, the basic model T0 neither revealed differences between the EDG and the NDG for the average time spent on the test nor for the changes in time-on-task over the course of testing. This was also the case for model T1. In T1, trait self-control and the trait self-control—ego-depletion interaction term were included as covariates at Level 2. As expected, students with high trait self-control tended to spend more time on the test than students with low trait self-control (*d* = 0.24), whereas a significant effect of the interaction between ego depletion and trait self-control on students’ time-on-task was not found.

**Table 2:** Level 2 effects of the multilevel logistic regression models for time-on-task.

Time-on-task as a logistic function (in *logtime*)									
		Model T0				Model T1			
		Estimate	*(SE)*	*p*	95% CI	Estimate	*(SE)*	*p*	95% CI
*Group (EDG in ref*. *to NDG)*									
Intercept	*β*_0*G*_	0.09	0.06	.150	[- 0.03, 0.21]	0.07	0.06	.264	[- 0.05, 0.19]
Slope	*β*_1*G*_	- 0.03	0.05	.461	[- 0.13, 0.07]	- 0.04	0.09	.703	[- 0.22, 0.14]
*Trait self-control (TSC)*									
Intercept	*β*_0*TSC*_	-	-	-	-	0.08	0.04	.054	[0.002, 0.16]
Slope	*β*_1*TSC*_	-	-	-	-	- 0.02	0.06	.705	[- 0.14, 0.10]
Group*TSC									
Intercept	*β*_0*G*_*TSC*_	-	-	-	-	- 0.01	0.07	.870	[- 0.15, 0.13]
Slope	*β*_0*G*_*TSC*_	-	-	-	-	- 0.03	0.09	.717	[- 0.21, 0.15]
*Level 2 Residuals*									
Intercept		0.11	0.02	<.001	[0.07, 0.15]	0.11	0.02	<.001	[0.07, 0.15]
Slope		0.14	0.04	<.001	[0.06, 0.22]	0.14	0.04	<.001	[0.06, 0.22]
**R**^**2**^_**Level 2**_									
Intercept		-				.05			
Slope		-				.01			

*Note*. EDG = Ego-Depletion Group; NDG = No Depletion Group; G = Group; TSC = Trait self-control; CI = confidence interval; R^2^_Level 2_ = proportion of reduction in variance on Level 2; All *p* values were two-tailed. For the sake of clarity, the fixed effects on Level 1 were omitted.

### Self-control and cognitive performance

**Fig 1:**
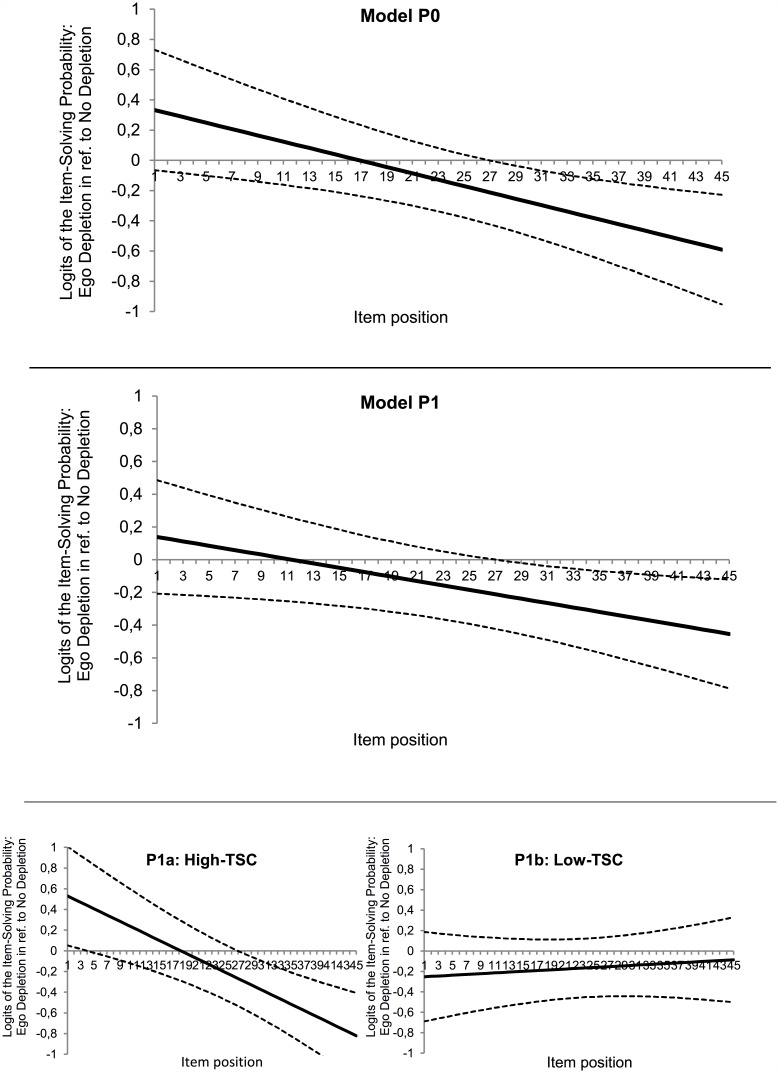
Ego-depletion-dependent performance decreases over the course of time. P0 and P1: Logits of the changing item-solving probabilities over the course of testing (Item Position 1–45) for students assigned to the ego-depletion group in reference to the no depletion group (solid line). The 95% confidence intervals (dashed lines) indicate that the probability of solving item 28 and any following item was significantly lower for the ego-depletion group. P1a and P1b: Decomposed effects of P1, separated for high trait self-control (TSC) students (P1a) and low-trait self-control students (P1b).

The average test performance and average performance change over the course of the MPSR test can be interpreted on the *logit metric*. Again, the results for the treatment variable of the models P0 and P1 (cf. Table 3) can be interpreted as effects for the EDG in reference to the NDG. The intercepts represent the EDG-students’ average test performance, whereas the slope effects indicate their performance changes over the course of testing in reference to students of the NDG. In the basic model P0, no significant differences were found between both groups’ average probabilities of solving all items. As expected, a significant decrease in the probability of solving the items was found over the course of testing for the EDG compared to the NDG (*d* = 0.65). This treatment effect was quite robust for the model P1. P0 in Fig 1 shows the logits of the decreasing item-solving probabilities (on the y-axis) from the first to the last item (on the x-axis) for the EDG in reference to the NDG. The probability of solving item 28 and any further items was significantly lower for the EDG (*β*_0*G*_ = - 0.23; *SE* = 0.11; *p* = .040; 95% confidence interval [CI] [- 0.44, - 0.01], odds ratio [OR] = 0.79) than for the NDG.

**Table 3:** Level 2 effects of the multilevel logistic regression models for mathematical performance.

Item solving probabilities as a logistic function (in *logits*)									
		Model P0				Model P1			
		Estimate	*(SE)*	*p*	95% CI	Estimate	*(SE)*	*p*	95% CI
*Group (EDG in ref*. *to NDG)*									
Intercept	*β*_0*G*_	- 0.12	0.11	.251	[- 0.34, 0.10]	- 0.16	0.11	.137	[- 0.38, 0.06]
Slope	*β*_1*G*_	- 0.92	0.33	.004	[- 1.57, 0.27]	- 0.59	0.28	.031	[- 1.14, 0.04]
*Trait self-control (TSC)*									
Intercept	*β*_0*TSC*_	-	-	-	-	- 0.06	0.06	.337	[- 0.18, 0.06]
Slope	*β*_1*TSC*_	-	-	-	-	0.03	0.17	.877	[- 0.30, 0.36]
Group*TSC									
Intercept	*β*_0*G_TSC*_	-	-	-	-	0.07	0.12	.563	[- 0.17, 0.31]
Slope	*β*_1*G_TSC*_	-	-	-	-	- 0.79	0.28	.004	[- 1.34, 0.24]
*Time-on-task*									
Intercept	*β*_0*Time*_	-	-	-	-	0.16	0.08	.047	[0.003, 0.32]
Slope	*β*_1*Time*_	-	-	-	-	- 0.82	0.24	.001	[- 1.29, 0.35]
Group*Time									
Intercept	*β*_0*G_Time*_	-	-	-	-	- 0.15	0.11	.175	[- 0.37, 0.07]
Slope	*β*_1*G_Time*_	-	-	-	-	0.04	0.32	.893	[- 0.59, 0.67]
TSC*Time									
Intercept	*β*_0*TSC_Time*_	-	-	-	-	- 0.08	0.05	.102	[- 0.18, 0.02]
Slope	*β*_1*TSC_Time*_	-	-	-	-	0.10	0.15	.518	[- 0.19, 0.39]
*Level 2 Residuals*									
Intercept		0.23	0.05	<.001	[0.13, 0.33]	0.21	0.05	<.001	[0.11, 0.31]
Slope		1.79	0.67	.007	[0.48, 3.10]	0.89	0.41	.029	[0.09, 1.69]
**R**^**2**^_**Level 2**_									
Intercept		-				.09			
Slope		-				.50			

*Note*. EDG = Ego-Depletion Group; NDG = No Depletion Group; G = Group; TSC = Trait self-control; CI = confidence interval; R^2^_Level 2_ = proportion of reduction in variance on Level 2; All *p* values were two-tailed. For the sake of clarity, the fixed effects on Level 1 were omitted.

To investigate the trait self-control—ego-depletion interaction effect on performance, we included trait self-control and the trait self-control—ego-depletion interaction term in model P1. Furthermore, as trait self-control (but not the ego-depletion manipulation) tended to be positively related to time-on-task (cf. T1), we included the average testing time, the ego-depletion—time-on-task and trait self-control—time-on-task interaction terms in P1 to control for test-taking effort (indicated by time-on-task) and to increase the test power.

In P1, the slope effect for the trait self-control—ego-depletion interaction became significant (*d* = 0.48). This means that depleted high-trait self-control individuals showed stronger performance decreases than depleted low-trait self-control individuals. P1a and P1b in Fig 1 represent the pattern of effects on the item responses of individuals scoring high (*M* + 1*SD*) and low (*M*– 1*SD*) on trait self-control.

The comparison of the trait self-control groups provides a visualization of the interaction effect between treatment conditions, trait self-control, and the item positions on the probabilities of solving the items correctly (controlling for item effects): Depleted high-trait self-control students (P1a in Fig 1) performed better on the first items but their probability of solving the items decreased more over the course of testing. The performance of depleted low-trait self-control students differed marginally from non-depleted low-trait self-control students (P1b in Fig 1).

In order to investigate the robustness of our results, we additionally extended the models by including the students’ cognitive ability test scores as a covariate in the models P0 and P1. In other words, we tested whether the results for P0 and P1 changed when also controlling for students’ cognitive abilities, which are known to be closely related to mathematical performance (e.g., [66]). In line with that findings, students’ cognitive abilities were positively related to the average performance in the MPSR test in P0 (i.e., the model that only included the ego-depletion manipulation variable) and P1 (i.e., the model that included all covariates given in Table 3; For P0 and P1 *β*_0*cog*.*abilities*_ = 0.32, *SE* = 0.05, *p* < .001, 95% CI = [0.22, 0.42], *d* = 0.66). Students’ cognitive abilities were found to have negative effects on changes in their MPSR performance in P0 (*β*_1*cog*.*abilities*_ = - 0.64, *SE* = 0.15, *p* < .001, 95% CI = [- 0.93, - 0.35], *d* = 0.45) and P1 (*β*_1*cog*.*abilities*_ = - 0.42, *SE* = 0.14, *p* = .002, 95% CI = [- 0.69, - 0.15], *d* = 0.29). However, despite the additional effects of the students’ cognitive ability test scores, our results revealed that all significant effects of ego depletion, trait self-control and their interaction on mathematical performance (as presented in Table 3) remained stable in both models. In addition, when controlling for students’ cognitive abilities, we found significant ego-depletion effects on the average mathematical performance in P0 (*β*_0*G*_ = - 0.18, *SE* = 0.09, *p* = .048, 95% CI = [- 0.36, - 0.004], *d* = 0.37) and P1 (*β*_0*G*_ = - 0.18, *SE* = 0.09, *p* = .046, 95% CI = [- 0.36, - 0.004], *d* = 0.37).

### Ego-depletion and fatigue effects

A repeated measures ANOVA on fatigue at the beginning versus the end of test session two, including the group as a factor, revealed a significant main effect for time (*M*_pre_ = 9.70, *SE* = 0.50, 95% CI = [8.69, 10.68] vs. *M*_post_ = 12.73, *SE* = 0.51, 95% CI = [11.71, 13.74]), *F*(1, 118) = 44.13, *p* < .001, $\eta_{p}^{2}$ = .27. However, no significant effect was found for the experimental manipulation *F*(1, 118) = 1.73, *p* = .190, $\eta_{p}^{2}$ = .02; and for the interaction between time and group on fatigue *F*(1, 118) = 0.70, *p* = .403, $\eta_{p}^{2}$ = .01. Thus, students’ perceived fatigue increased significantly but the pattern did not differ between the EDG and the NDG.

### Additional analyses

To rule out the possibility that the ego-depletion manipulation induced differences in students’ reported affect, test-taking motivation, and invested test-taking effort, we investigated ego-depletion effects on these potential confounding variables. Furthermore, we investigated whether these variables were related to trait self-control and the trait self-control—ego-depletion interaction. As presented in Table 4, linear regression analyses showed that neither ego depletion nor the trait self-control—ego-depletion interaction was a significant predictor of any of these variables. However, trait self-control turned out to be positively related to positive affect, test-taking motivation, and invested test-taking effort.

**Table 4:** Linear regression models for predicting control variables on ego depletion, trait self-control and the interaction between both variables.

Variable	Positive Affect(*α* = .85)			Negative Affect(*α* = .75)			Motivation: General(*α* = .79)			Motivation: Math(*α* = .69)			Test Effort -		
	B	*SE*	β	B	*SE*	β	B	*SE*	β	B	*SE*	β	B	*SE*	β
Ego Depletion	1.39	1.23	.10	0.19	0.78	.02	- 0.45	0.53	- .08	- 0.39	0.40	- .08	- 0.24	0.35	- .06
Trait self-control (TSC)	1.91	0.85	.28*	0.09	0.54	.02	0.66	0.37	.23	0.70	0.27	.32*	0.53	0.24	.28*
Ego Depletion * TSC	0.15	1.24	.02	- 0.45	0.79	-.07	0.12	0.54	.03	- 0.30	0.40	- .09	- 0.21	0.35	- .08

*Note*. *α* = Cronbach’s reliability coefficient.

*** *p* ≤ .001,

** *p* ≤ .01

* *p* ≤ .05

Furthermore, to rule out the alternative explanation that high-trait self-control students invested more effort on the initial transcription task, we correlated trait self-control with both the number of written words (*r* = .02, *p* = .901) and the number of errors in the transcription task (*r* = -.11, *p* = .414) for the EDG. Both correlations were not significant; thus, the effects of our main results cannot be explained by a higher level of exhaustion experienced by high-trait self-control students in the ego-depletion task.

Finally, we investigated whether the differences between the ego-depletion-induced performance patterns for the high-trait self-control group (*N*_EDG_ = 11; *N*_NDG_ = 9) and the low-trait self-control group (*N*_EDG_ = 11; *N*_NDG_ = 10) were due to differences in both groups’ average performance levels. Even when controlling for the students’ cognitive abilities, a 2 × 2 ANCOVA on the MPSR score did not yield a significant interaction between the ego-depletion (EDG vs. NDG) and the trait self-control (high trait self-control vs. low trait self-control) conditions: *F*(1, 36) = 0.542, *p* = .466, $\eta_{p}^{2}$ = .015. Neither the main effect of ego depletion, *F*(1, 36) = 2.214, *p* = .145, $\eta_{p}^{2}$ = .058, nor the main effect of the trait self-control group, *F*(1, 36) = 0.016, *p* = .901, $\eta_{p}^{2}$ = .000, were significant. Thus, the ego-depletion intervention did not affect either trait self-control group’s average performance, but did affect how their performance changed over the course of the test.

## Discussion

The aim of the present study was to investigate how ego depletion and trait self-control affect students’ invested test-taking effort and performance over the course of an ecologically valid testing situation. By combining the sequential-task paradigm with multilevel modeling our results underline the importance of investigating not only average ego-depletion effects and their relation to trait self-control, but also progressive effects over time.

First, we will discuss our findings on the relations between ego depletion and motivational variables. In contrast to our expectations, the EDG and NDG did not differ significantly in any of the time-on-task parameters, the reported test-taking motivation, or the invested effort. According to the process model of Inzlicht and Schmeichel [8], which suggests that exerting self-control progressively undermines the effort invested when working on tasks that require self-control, the EDG should have spent less time-on-task, indicating less invested test-taking effort, than the NDG; this was not the case. Furthermore, the self-reported effort invested and test-taking motivation should have been lower for the EDG than for the NDG, which was also not the case in the present study. These results indicate that the ego-depletion intervention did not necessarily increase the students’ urge to reduce their test-taking effort or even to quit working on the test. Thus, our findings rule out the frequently raised alternative explanation for ego-depletion effects that suggests that participants feel that they have done enough work for the experiment after the ego-depletion intervention and abandon their obligation to invest effort on the subsequent task [67]. However, it can be assumed that working on mathematical problems is part of the daily routine for tenth graders; therefore, participants might have considered it worthwhile to practice their mathematical skills by investing time and effort on solving the items over the course of testing, regardless of whether they previously had to exert self-control or not. Thus, contrary to the findings (e.g., [32, 33]) that ego depletion undermines the persistence to work on ‘artificial’ tasks (e.g., unsolvable anagrams), our results raise the question of whether ego depletion negatively affects individuals’ motivation to work on ecologically valid tasks that require self-control and with which they are also confronted in their everyday life. Our results suggest that this is not the case, but further research is required to investigate the effects of ego depletion on students’ achievement motivation in school or in academic contexts.

Second, whereas motivational variables (motivation, invested test-taking effort, time-on-task) were not affected by the ego-depletion manipulation, the mathematics performance of the EDG decreased more strongly over time, indicating that the ego-depletion intervention undermined effective cognitive processing during problem solving. In the second half of the MPSR test, the EDG performed at a consistently lower level than the NDG (see Fig 1, P0), indicating that the self-control required in order to keep attention focused during information processing wanes over time (cf. [10]). Even when controlling for students’ cognitive abilities, the ego-depletion-induced performance decreases remained stable. Furthermore, in line with the findings of Bertrams et al. [17], significant ego-depletion effects on the average performance score in mathematics were found in both models (P0 and P1) when considering the students’ cognitive abilities as a covariate. We speculate that the ego-depletion-induced performance decreases in the MPSR test seem to depend on subconscious cognitive processes, such as fluctuations in executive control (cf. [5]) or attention shifts away from cues signaling the need for self-control and toward cues signaling rewards, as proposed by the process model of Inzlicht and Schmeichel [8]. Whereas the process model is a viable option for investigating the underlying mechanisms of self-control in future research, it has to be noted that the ego-depletion-dependent performance decreases we found can also be interpreted by referring to the strength model of self-control [1]. Based on the strength model, it can be assumed that the finite self-control resource became increasingly depleted during the ego-depletion manipulation as well as during the mathematics test. As a result, the students of the EDG had fewer resources available than the NDG for controlling their attention in the second half of the test; therefore, their performance decreased. Whichever model for explaining ego depletion is preferred, our results are in line with the findings that ego depletion increases peoples’ distractibility (e.g., [68, 69]), whereby working-memory operations become disrupted (cf. [5]), leading to performance decrements in problem-solving and reasoning tasks [4, 15]. Interestingly, in our study, no differences were found between the self-evaluation of fatigue of the EDG and NDG. Therefore, the stronger performance decrements in the EDG could not be explained by ego-depletion-induced fatigue effects. Whereas fatigue is assumed to be a candidate for explaining the ego-depletion phenomenon [10], our findings contradict this assumption to a certain extent. However, we only asked the participants about their physical fatigue (e.g., feeling tired) and not about their mental fatigue (e.g., feeling mentally exhausted). The latter seems to be negatively related to goal-directed attention [70]. Therefore, in line with Kaplan and Berman [71], who indicated that directed attention seems to play a major role in successful self-control, we suggest that future research should investigate the interplay between ego depletion and attention over the course of time (cf. [2]) in greater detail, while also considering mental fatigue as a covariate.

Finally, we will focus on our findings concerning the relations between trait self-control, ego depletion and their interaction on students’ test-taking motivation and cognitive performance. Students with high trait self-control tended to invest more time-on-task throughout testing and reported higher levels of test-taking motivation and effort than students with lower trait self-control. This suggests that high trait self-control seems to support students’ test-taking engagement [45] and might be necessary to initiate controlled processing for problem solving and reasoning. Interestingly, depleted high-trait self-control individuals showed stronger performance decrements over the course of testing than non-depleted high-trait self-control students. For the low-trait self-control group, no differences were found between the performance changes in the EDG and the NDG. This result is similar to the counterintuitive trait self-control—ego-depletion interplay reported by Imhoff et al. [9]. In their article, the authors assume that high-trait self-control individuals avoid self-control requiring situations in their daily life more often; therefore, they might have fewer self-control strategies available for dealing with the demands of ego-depletion experiments. As a consequence high-trait self-control individuals show stronger ego-depletion effects. Our results might extend the explanation for the counterintuitive trait self-control—ego-depletion interplay.

On the one hand, as proposed by Imhoff et al. [9], high-trait self-control individuals might have fewer self-control strategies for dealing with tasks that require attention control. On the other hand, we found that high-trait self-control students were highly motivated and invested higher levels of effort and time to solve the MPSR test. Taken together, the greater effort that depleted high-trait self-control students invested to solve the items of the MPSR test might have required more mental capacities and more efficient self-control strategies for keeping the attention focused on the items’ content over the course of testing. Thus, when depleted but highly motivated high-trait self-control students diminished their mental capacities during the initial ego-depletion manipulation, attention regulation and mental processing might have become more and more error-prone, resulting in stronger performance decrements in the MPSR test. Our speculations imply that high-trait self-control students might have also invested more effort when working on the ego-depletion manipulation task, resulting in a stronger ego-depletion induction. Therefore, we conducted several control analyses and were thereby able to rule out this alternative explanation. In contrast, depleted low-trait self-control students who were less motivated to work on the MPSR test than high-trait self-control students did not differ in their performance trajectories from low-trait self-control students in the NDG. In both the EDG and NDG, low-trait self-control students did not seem to become over-involved when working on the MPSR test, and this resulted in them having higher levels of mental endurance, which was especially needed for working on the second half of the test.

The counterintuitive trait self-control—ego-depletion interplay found in our study can be illustrated by referring on Baumeister’s [1] muscle metaphor (i.e., finite self-control resources briefly become depleted like a muscle after physical exercise). Relating this idea to our results, high-trait self-control individuals exerted their self-control strength during the ego-depletion intervention task and the high levels of test-taking effort they invested when working on the subsequent MPSR test might have consumed high levels of self-control resources. As a consequence, their performance decreased at a faster rate over the course of testing, compared to depleted low-trait self-control students, who conserved their resources and showed less test-taking motivation. In other words, the depleted high-trait self-control individuals might have been more motivated to perform well in resource-consuming cognitive tasks than the depleted but less motivated low-trait self-control individuals. It has to be noted that the scientific utility of Baumeister’s postulated self-control resources has been questioned by ego-depletion researchers (e.g., [2, 8, 10, 21]) because this concept is difficult to falsify in empirical research [72].

Alternatively, in line with the process model of self-control of Inzlicht, Schmeichel and Macrae [2], the counterintuitive trait self-control—ego-depletion interaction effect on the performance decrements in mathematics could be explained as follows: Due to the higher test-taking engagement shown by high-trait self-control individuals, it might have become more and more effortful for them to keep their attention focused during cognitive processing, especially when they had already exerted self-control during the initial ego-depletion intervention task. As a result, stronger attentional shifts toward test-irrelevant cues and, therefore, increasing cognitive interruptions might explain the larger performance decreases we found for depleted high-trait self-control individuals.

Hence, our findings are compatible with both theoretical explanations (i.e., finite self-control resources vs. attentional shift), so that we cannot favor one explanation over the other based on the present data. We suggest further research to investigate ego-depletion-dependent shifts of attention, the corresponding cognitive interruptions, and the resulting performance decrements over the course of time.

One limitation of the present study may be that we did not investigate whether depleted individuals, compared to non-depleted participants, experienced the mathematics test as more and more aversive or whether they experienced an increasing urge to quit the test. Therefore, it would be useful for future research to investigate whether depleted individuals experience achievement testing as being more aversive than non-depleted participants. On the other hand, our study did not find any differences between EDG- and NDG-students’ average motivation and its changes (test-taking time, self-reported test-taking motivation, and the invested effort). Therefore, it can be speculated that increasing urges to quit the test would probably have been reflected in ego-depletion-dependent time-on-task decrements, which was not the case.

To sum up, modeling progressive ego-depletion patterns over the course of time while also considering covariates such as trait self-control seems to be a useful approach for future self-control research, because it facilitates a new perspective on the ego-depletion phenomenon and its relation to various covariates. To the best of our knowledge, the results of our study are the first to provide evidence that the self-control invested in order to keep performance at a consistently high level wanes over time [10].

## Conclusion

In conclusion, our study provides first insights into the effects of trait self-control, ego depletion, and their interplay on time-on-task and cognitive performance trajectories over the course of achievement testing. With regard to our findings, modeling ego-depletion-dependent changes in time-on-task and cognitive performance revealed that ego depletion did not affect students’ test-taking motivation (i.e. time-on-task, self-reported test-taking motivation, and invested test-taking effort) but led to performance decrements over the course of time. Additional analyses revealed that our results could not be alternatively explained by fatigue effects. In contrast, trait self-control was positively related to the engagement and effort individuals invested to solve the items. Due to the higher test-taking effort that high-trait self-control individuals invested, their performance decrements were stronger when they were in a state of ego depletion compared to low-trait self-control individuals. Therefore, individuals with higher levels of trait self-control seem to deplete their self-control at a higher level due to their more intense use of the mental capacities needed for attention control and information processing.

Taken together, our results provide evidence that self-control wanes over time [10] and that ego depletion seems to be a real phenomenon with which individuals might be confronted in everyday life achievement situations. We have demonstrated some benefits of modeling progressive depletion patterns over time instead of considering only the average performance or time-on task; this may be an effective approach for future ego-depletion research. Thus, the recommendation of Inzlicht and Schmeichel [8] to investigate self-control processes over time seems a promising option for gaining deeper insights into the underlying mechanisms of self-control.
